# Neutron visualization of inhomogeneous buried interfaces in thin films

**DOI:** 10.1038/s41598-018-37094-5

**Published:** 2019-01-24

**Authors:** Kenji Sakurai, Jinxing Jiang, Mari Mizusawa, Takayoshi Ito, Kazuhiro Akutsu, Noboru Miyata

**Affiliations:** 1 National Institute for Material Science, 1-2-1, Sengen, Tsukuba, Ibaraki 305-0047 Japan; 2 University of Tsukuba, 1-1-1, Tennodai, Tsukuba, Ibaraki 305-0006 Japan; 3 Comprehensive Research Organization for Science and Society (CROSS), Tokai, Ibaraki 319-1106 Japan

## Abstract

When designing some functions in thin film systems, one of the key concepts is the structure of the constituent layers and interfaces. In an actual system, the layers and interfaces are often inhomogeneous in different scales, from hundreds of microns to several nanometers, causing differences in properties, despite very similar average structures. In this case, the choice of the observation point is critical to clarify the problem. Another critical aspect is the identification of these points by surveying the entire inhomogeneous thin film system. This article presents a description of a novel promising solution that is suitable for nondestructive visualization of inhomogeneous buried layers and interfaces in thin films. Such observations have been impossible until now. In this investigation, a unique extension of neutron reflectometry is proposed. While conventional neutron reflectivity just gives average depth-profiling of the scattering length density of layered thin films, the present method provides full picture of the inhomogeneity. In general, achieving a high spatial-resolving power for neutron scattering is not straightforward because the neutron counts become fairly limited at the sample or the detector position when the beam size is reduced. As a result, XY scanning of a sample with a small neutron beam is fairly difficult because of the required long measurement time. To address these issues, new concepts have been introduced for neutron reflectivity. The proposed method uses a wide beam instead of reducing the beam size. In addition, it measures the projection reflection profile instead of the total integrated intensity. These profiles are collected at a set of different in-plane angles. Similar to computed tomography, it is possible to obtain the specimen’s two-dimensional (2D) neutron reflectivity distribution as one image. Because the spatial resolution is limited by the detection method, a Hadamard coded mask is employed to measure the reflection projection with only 50% loss of the primary neutron intensity. When the time-of-flight (ToF) mode is used for the neutron experiment, one can obtain many images as a function of ToF, i.e., the wavevector transfer. Such series of images can be displayed as a video. This indicates that the neutron reflectivity profiles of local points can be retrieved from the above video images. This paper presents the first report on the development of neutron reflectivity with imaging capability, and the analysis of local points in inhomogeneous layered thin-films without utilizing a small neutron beam. In the present work, the feasibility of the proposed method with approximately 1 mm spatial resolution was examined. In addition, further improvements of the approach are discussed. It is anticipated that this technique will facilitate new opportunities in the study of buried function interfaces.

## Introduction

With the rapid development of nanotechnology, numerous microdevices have been prepared as buried structures in multilayered ultra-thin films^1–7^. In general, subsequent layering can exert an influence on the structure by chemical diffusion^8,9^, and therefore, precise knowledge on the final form of these structures is extremely important. Furthermore, the layers and interfaces are quite often inhomogeneous in different scales, from hundreds of microns to several nanometers. This causes differences in the properties of these thin films, in spite of very similar average structures. Visualization of the buried layers and interfaces would be of great benefit in clarifying the physical reasons for the variation in the properties and/or functions. Modern sophisticated analytical techniques can be used if the structures are directly exposed, or if the outer layers are extremely thin. Because thin films are typically covered with thick protection layers, destructive inspection is often the only viable option. Cross-sectional specimens can be studied using transmission electron microscopy and other microscopy techniques. In this case, the choice of the observation position  is critical in clarifying the problem. Another equally important aspect is the identification of these points by surveying the entire inhomogeneous thin film system.

This paper reports some initial success in the visualization of inhomogeneous layers and interfaces in thin films using neutrons. This approach can facilitate nondestructive probing of deeply buried structures beneath a thick layer. The proposed method is an extension of neutron reflectometry, which allows for the depth profiling of the scattering length density in layered systems^10–12^. The method is powerful because it has a few unique advantages in detecting small changes in the thickness and density of a layer, as well as the roughness of surfaces and interfaces. In comparison with X-rays, neutrons have scientifically interesting features^13^ such as extraordinary penetrating power, sensitivity to magnetism, as well as capability of distinguishing organic function groups by deuteration. However, it should be noted that the main problem associated with conventional neutron reflectometry is the lack of spatial resolution. In addition, the technique usually requires a large sample for measurement. Because practical functional thin films are essentially inhomogeneous, local features in the surface and interfaces of these films should not be neglected. To achieve a high spatial resolution, a possible approach is to use a small beam. In this regard, the development of neutron focusing optics is important. However, achieving XY scanning with a reduced neutron beam is not straightforward because the neutron counts are fairly limited at the sample or the detector position. This leads to an inordinately long measuring time for many collection points. In addition, it should be noted that the spatial resolution in the grazing-incidence geometry is much worse than one would expect for a given beam size because of the elongated footprint of the neutron beam on the sample. For example, when the grazing angle is 0.3°, the beam size in the beam propagation direction is approximately 190 times greater than the original size. To probe a small area of the sample, some additional optics or the truncation of the beam is necessary. Until now, because of the associated difficulties, neutron reflectivity imaging has not been employed. A breakthrough has been identified in the use of projection-type imaging with a large beam size (rather than XY scanning-type imaging with a smaller beam size). The new method measures the profile of the reflection projection from inhomogeneous samples, instead of integrating the reflected neutron intensity. After collecting many such projections while rotating the in-plane angle, an image reconstruction scheme similar to that used for computed tomography, can be used. The authors have tested the aforementioned reflectivity imaging method using monochromatic X-rays with image detectors such as CCD cameras^14–18^. In the past, the image reconstruction scheme was applied to other X-ray methods, such as grazing-incidence small angle X-ray scattering^19^ and X-ray diffraction imaging^20^.

## Results

### Comparison between conventional neutron reflectometry and the proposed neutron reflectivity imaging

**Figure 1:**
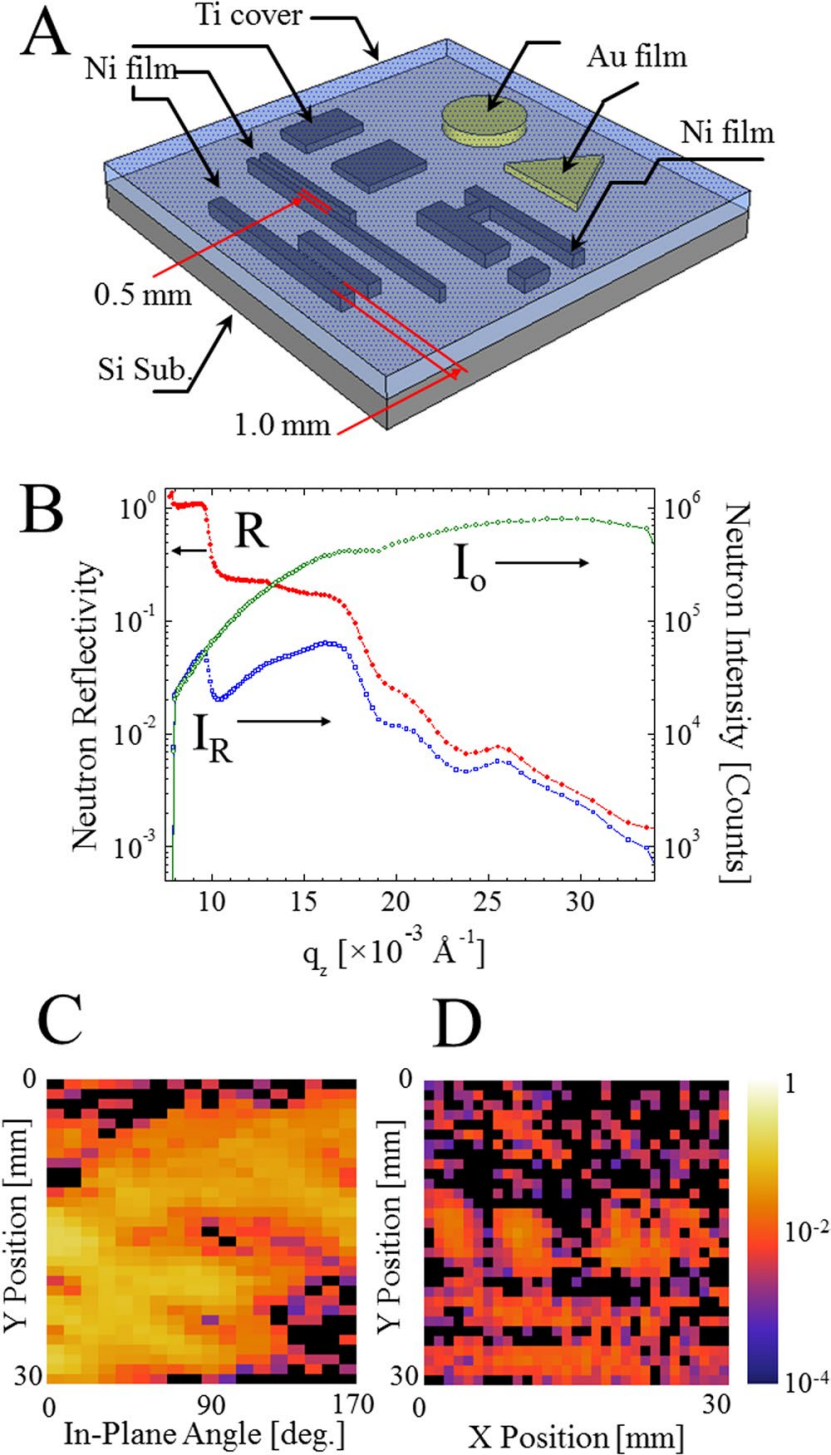
Schematic of the heterogeneous model sample composed of shaped gold thin films (the short cylinder and the pentahedron, thickness of 140 Å and 400 Å) and patterned nickel thin films (polygons, thickness of 500 Å, 750 Å, and 1000 Å) covered by a homogeneous titanium thin layer (of approximately 1320 Å). (**A**) Conventional neutron reflectivity data for the patterned sample (schematically shown in **A**) taken while neglecting its inhomogeneity (measured at the in-plane angle of 0°). Io spectra acquired without sample (green open circle ○), I_R_ spectra (integrated neutron reflection intensity, blue open square ), and R (the ratio of the reflected intensity and Io intensity, red closed circle ). (**B**) Sinogram of the patterned sample (schematically shown in **A**) for q_z_ = 14.8 × 10^−3^ Å^−1^. The sinogram is a collection of the reflection projection as a function of the in-plane rotation angle. Because the experiments are the ToF mode, a series of sinogram images are collected for many q_z_. (**C**) An XY reflectivity image of the patterned sample (schematically shown in** A**) for q_z_ = 14.8 × 10^−3^ Å^−1^. It is obtained by the image reconstruction of the sinogram (shown in **C**). The image reveals the position and the shape of the buried metallic layers. (**D**)  Finally obtained data are a video collecting the XY reflectivity images (such as **D**) as a function of q_z_. One example of such video is given in the supplementary materials.

In the present research, a patterned multilayer sample, Ti/Au-Ni/Si, schematically shown in Fig. 1A, was studied to examine the feasibility of the proposed neutron reflectivity imaging method. It is sometimes possible to employ the conventional neutron reflectometry without noticing the inhomogeneity of the sample to be studied. Figure 1B shows such an “average” reflectivity profile for the sample of Fig. 1A. In conventional neutron reflectometry, the profile R is usually expressed as a function of the wavevector transfer ${{\rm{q}}}_{{\rm{z}}}=\frac{4{\rm{\pi }}\,\sin \,{\rm{\theta }}}{{\rm{\lambda }}}$, where the subscript z is the in-depth direction in the sample frame. This demonstrates that only the wavevector transfer along the in-depth direction exists in the case of specular reflection. The angle θ is the neutron grazing incident angle and λ is the de Broglie wavelength of the neutrons. In this study, to obtain an entire neutron reflectivity profile R(q_z_), the time-of-flight (ToF) measurement was performed at the fixed θ/2θ = 0.35/0.7° with a white pulsed neutron beam. Essentially, the same data can be obtained by a θ/2θ scan with a monochromatic continuous neutron beam. For the ToF measurement, the neutron wavelength λ can be written as $\lambda =\frac{h}{p}=\frac{ht}{mL}$, where *h* is Planck’s constant, p is the neutron’s momentum, t is the ToF, m is the neutron’s mass, and L is the source-to-detector distance. Therefore, after normalization using the spectral distribution of the primary white neutron beam (displayed as Io in Fig. 1B), R(q_z_) is obtained. In Fig. 1B, some clear steps can be observed at 10 × 10^−3^ Å^−1^ and 18 × 10^−3^ Å^−1^, which correspond to the critical q_z_ for silicon and nickel, respectively, in addition to some interference fringes from approximately 20 × 10^−3^ Å^−1^ to 34 × 10^−3^ Å^−1^. The data are the average of the entire area. The number of neutron counts for the reflection is lower than 10^5^, and the reason is a quite shorter accumulation time of 272 sec, which is set for the present imaging experiment. When the sample is not uniform, R_qz_(x, y) should be considered rather than the “average R(q_z_)”. Clearly, XY scanning of the sample is a direct way to obtain R_qz_(x, y), although unfortunately it is not very practical, as previously mentioned. In the present research, the measurement of neutron reflection projection as a function of q_z_ and the in-plane angle of the sample φ is proposed. Mathematically, the reflection projection P_φ,qz_(u) can be expressed as the integrated reflection intensity profile along the neutron forward direction according to the Radon transform^21^ as follows:

$${{\rm{P}}}_{{\rm{\phi }},{\rm{qz}}}({\rm{u}})={\int }_{-\infty }^{+\infty }\,{{\rm{R}}}_{{\rm{qz}}}(\mathrm{ucos}{\rm{\phi }}-\mathrm{vsin}{\rm{\phi }},\,\mathrm{usin}{\rm{\phi }}+\mathrm{vcos}{\rm{\phi }})\mathrm{dv},$$

where u is the projection position (perpendicular to the neutron forward direction), v is the position in the neutron forward direction, and R_qz_(x, y) is the neutron reflectivity at the local position (x, y). When P is expressed as P_qz_(φ, u), it is called a sinogram, while P_φ_(q_z_, u) is called a reflectogram. Figure 1C shows the experimentally obtained sinogram (at q_z_ = 14.8 × 10^−3^ Å^−1^) for the patterned sample shown in Fig. 1A. Similar to computed tomography, it is possible to obtain R_qz_(x, y) from the experimentally obtained sinogram. By applying image reconstruction based on the filtered back projection (FBP) method^21–24^, the XY reflectivity image shown in Fig. 1D was obtained. In Fig. 1D, good contrast is evident, and the features correspond to the pattern shown in Fig. 1A. In the present research, the ToF mode is used, and therefore, multiple sinograms are obtained instead of just one (in the present case, the number is 102, although it depends on the integration interval used for the ToF measurement). Therefore, the finally obtained data were a XY reflectivity video as is given in the supplementary materials. In short, while conventional neutron reflectometry facilitates a single profile including “average” layered structure information, the proposed neutron reflectivity imaging provides a set of real space images, which makes q_z_ dependent on the inhomogeneity of the layered structure in the sample.

### Hadamard encoding and decoding of reflection projection

**Figure 2:**
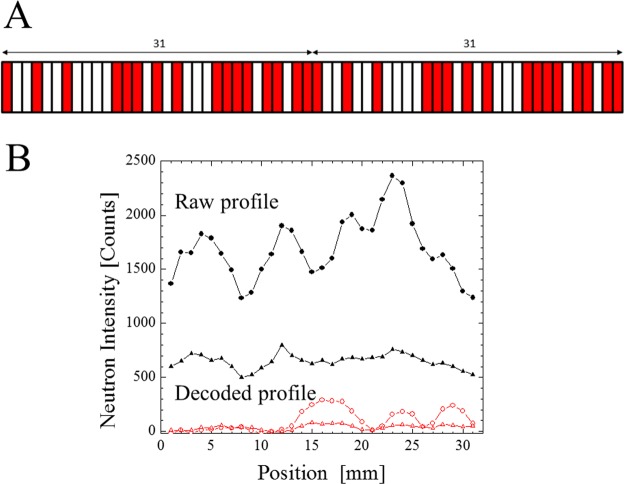
Schematic of Hadamard mask (31 slots, 2 repetitions, negative pattern). The colored and white parts are closed (with cadmium) and open (without cadmium), respectively, which mathematically correspond to +1 and −1 in the matrix shown in Eq. (1), respectively. The width of each slot is 1 mm. (**A**) Example of raw (black closed triangle ▲ and circle ●) and decoded (red open triangle  and circle ) profiles for the patterned sample (schematically shown in Fig. 1A) at q_z_ = 10.54 × 10^−3^ Å^−1^ (closed ▲ and open  triangles), and q_z_ = 14.8 × 10^−3^ Å^−1^ (closed ● and open  circles) (**B**).

The next important issue is a practical method to measure the reflection projection profile, P_φ,qz_(u) with sufficient spatial resolution. The spatial resolution is mainly limited by the pixel size of the image detector when the angular divergence of the neutron beam is optimized by the slit system at the beamline, as is the case for the present research. Given that the development of high-resolution one-dimensional/two-dimensional (2D) neutron detectors is still a significant challenge, alternative approaches were considered during the investigation. The proposed method involves scanning a Hadamard coded mask^25–28^ placed in front of a point detector (no pixels, no spatial resolution) such as a ^3^He detector. It is then apparent that P_φ,qz_(u) can be obtained by simply scanning a narrow slit placed in front of the ^3^He detector. However, the neutron intensity measured at each point would be relatively weak, leading to an impractically long measuring time. The key point in the implementation of the Hadamard coded mask is that the opening of the mask should be approximately 50%. Therefore, the counting statistics are not lost even when the opening slot size is small. Although Hadamard coded masks have not been employed in the neutron scattering research until now, the authors were able to validate the utility of obtaining neutron projection (reflectogram) through preliminary tests using a 15-slot Hadamard coded mask^25^. In the present research, a 31-slot Hadamard coded mask (as shown in Fig. 2A) was prepared and employed for the first neutron imaging experiment. As the original intensity profile (y_1_, y_2_, y_3_, y_4_, … y_31_) is encoded as (Y_1_, Y_2_, Y_3_, Y_4_, …Y_31_) by scanning the Hadamard coded mask with steps corresponding to the slot size, the relation is expressed as1

The matrix is 32 × 32, and the first line and the first row are filled by one. The 32 × 32 part represents the scanning condition of the Hadamard mask, corresponding to Fig. 2A. The transparent slot is represented by minus one, while the beam blocking slot represented by is plus one. Here we set the total sum of y_1_, y_2_, y_3_, y_4_, … and y_31_ as -y_0_. Because (Y_1_, Y_2_, Y_3_, Y_4_, …Y_31_) is obtained in the experiment, the original intensity profile (y_1_, y_2_, y_3_, y_4_, …y_31_) can be calculated by using this linear relationship. This corresponds to the decoding process, which uses the same matrix with a multiplication factor, for example −2/(N + 1) instead of −1/2, where N = 31. Figure 2B shows some decoding examples of experimentally obtained raw profiles for some specific q_z_ (10.5 × 10^−3^ Å^−1^ and 14.8 × 10^−3^ Å^−1^) taken at the in-plane angle of 0°. The decoded 3 peaks (approximately 17 mm, 23 mm, and 29 mm positions) correspond to the strong neutron reflection caused by the nickel coated parts. A weak part (approximately 6 mm position) corresponds to the gold-coated region. Because the in-plane angle is 0°, the projection direction is from left top to right bottom in Fig. 1A. While the number of decoded neutron counts are lower than 300, the experimentally observed encoded neutron counts are roughly 10 times. The Hadamard encoding always measure nearly 50% of reflections from different parts of the sample, and this greatly helps in reducing the statistical error.

### Reflectograms

**Figure 3:**
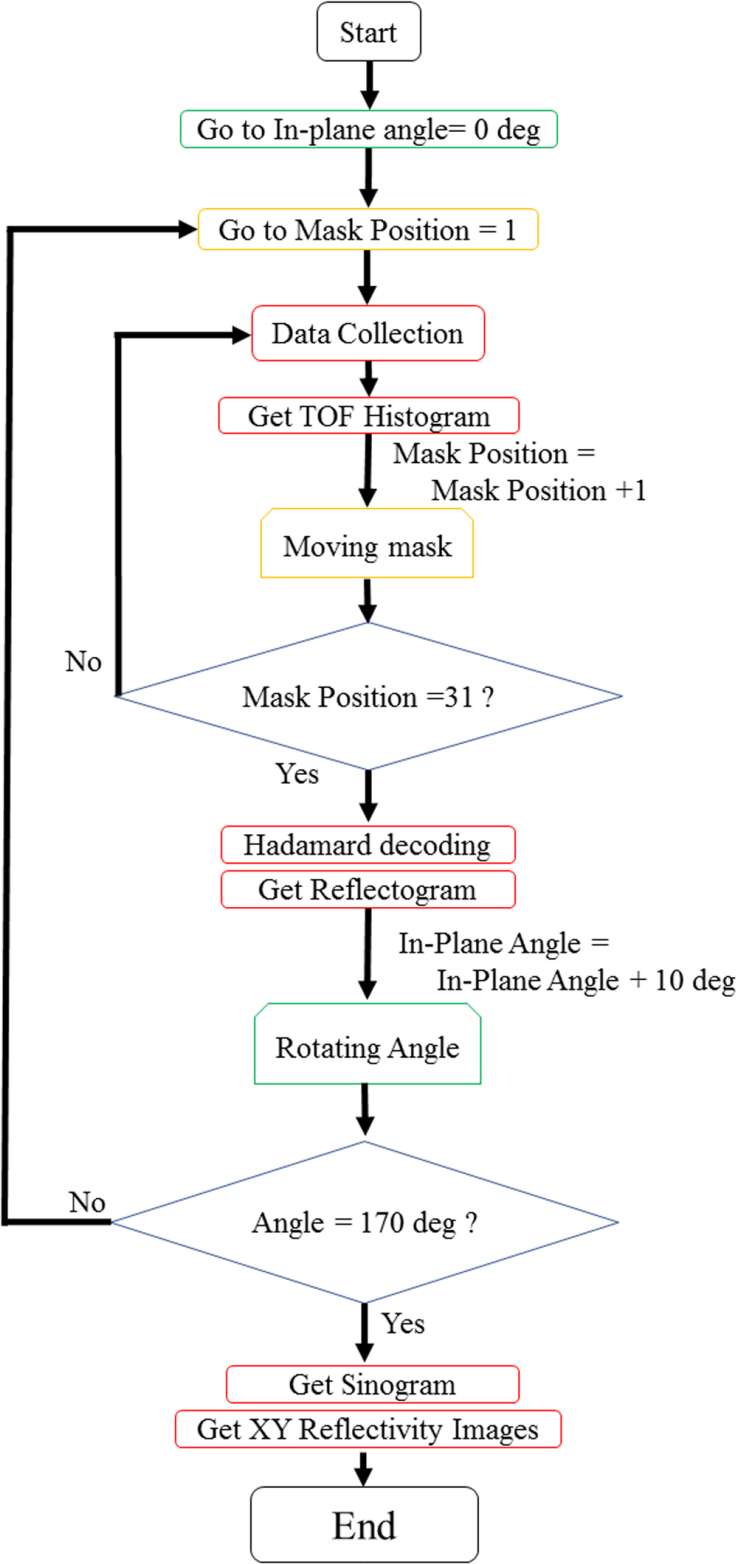
A flowchart of the measurement and the analysis in the proposed neutron reflectivity imaging. Green and orange squares correspond to the motion of stages (linear stage for the Hadamard coded mask and the in-plane rotation stage, respectively). Red squares correspond to the data handling.

**Figure 4:**
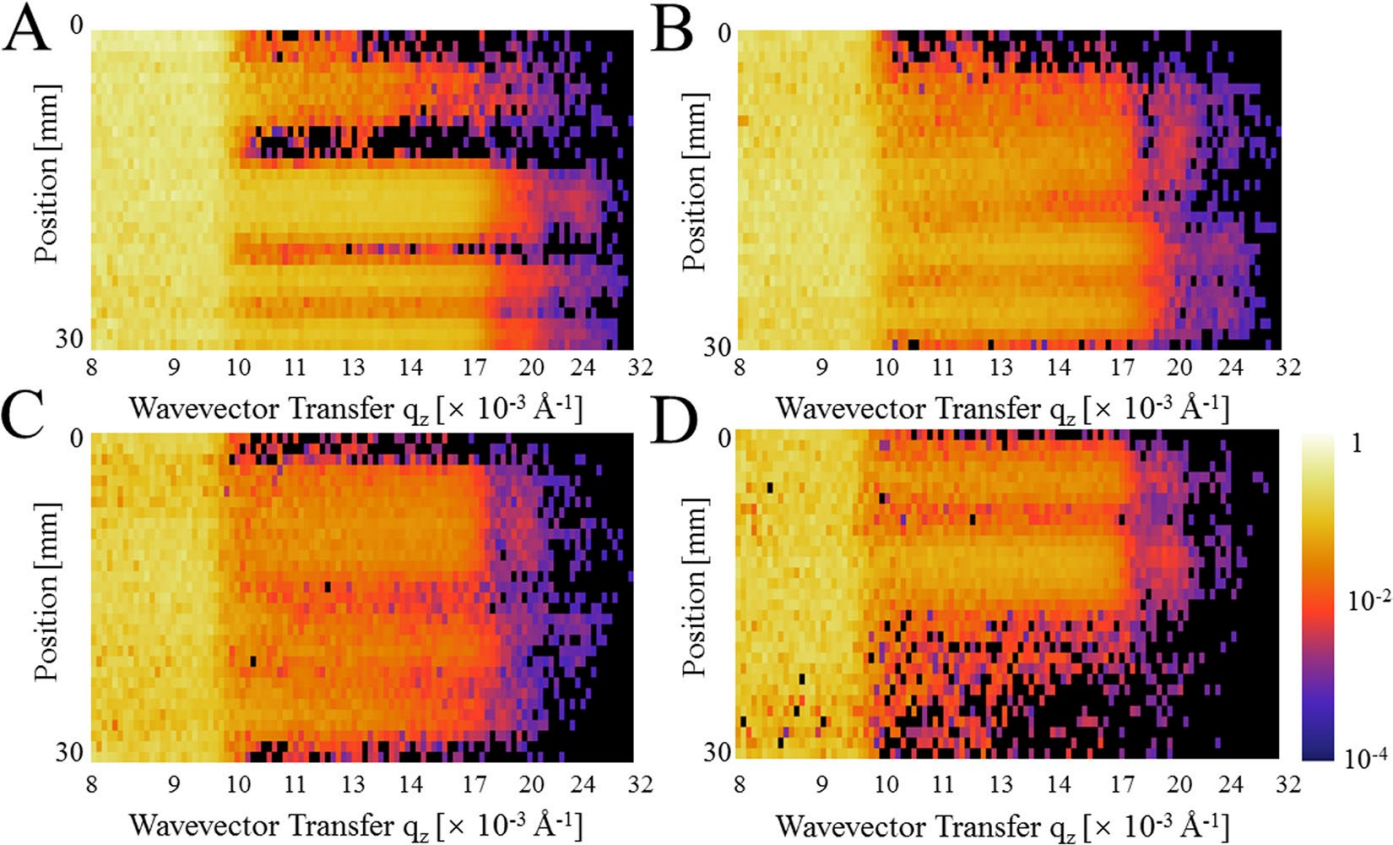
Neutron reflectograms for the patterned sample (schematically shown in Fig. 1A) taken at the in-plane angles of 0° (**A**), 60° (**B**), 120° (**C**), and 160° (**D**). The reflectogram shows the reflection projection as a function of q_z_. The images are displayed in the common logarithmic color scale. Note that the q_z_ axis of the image is not linear as the data are handled as a function of ToF, for which the bin width is a constant at Δt = 0.3 ms. In the present research, the data were taken for 18 in-plane angles.

As previously explained, the neutron intensity profile can be obtained by sliding the coded mask with a step corresponding to the slot size. In this case, 31 measurements are necessary because the number of slots in the mask is 31. The same measurements are repeated for different projection angles. Figure 3 shows the flowchart of the measurement process. Because 18 different projections are collected in the present work, the number of datasets becomes 31 × 18 = 558. Each dataset are ToF spectra and can be described as the neutron counts vs. q_z_ plot. Figure 4 shows the reflectogram, P_φ_(q_z_, u), for the different in-plane angles of 0°, 60°, 120°, and 160°. The data are obtained by decoding the raw profile and then normalizing with the Io spectra (shown in Fig. 1B). The flat total reflection region can be clearly identified for values lower than q_z_ = 9~10 × 10^−3^ Å^−1^. Another region of high contrast in the neutron reflectivity data is visible at approximately q_z_ = 18 × 10^−3^ Å^−1^, which corresponds to the critical q_z_ of nickel thin film. Although the literature value for the critical q_z_ for nickel is 22 × 10^−3^ Å^−1^, based on independent neutron reflectivity measurement of uniform thin films, it has been confirmed that the utilized sputtered thin nickel film exhibit a lower value. For the higher q_z_ part, the images exhibit some interference fringes. The critical q_z_ of gold (14 × 10^−3^ Å^−1^) is not obvious in the reflectogram, probably because the thicknesses of the gold layers are too thin to induce sharp reflectivity changes.

### Sinograms and image reconstruction

**Figure 5:**
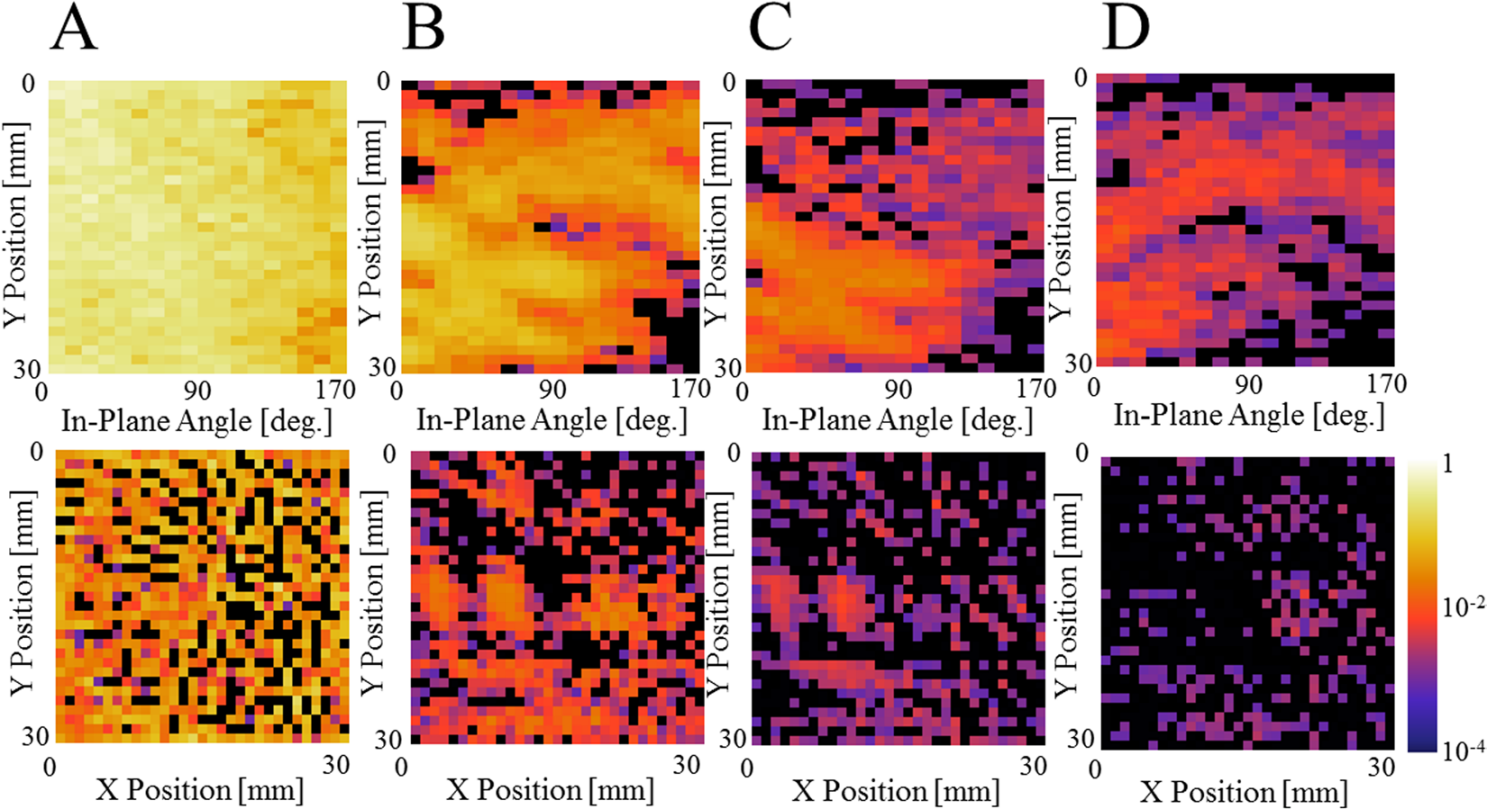
Neutron reflectivity sinogram (upper images) and the XY reflectivity images (lower images) of the patterned sample (schematically shown in Fig. 1A) for q_z_ = 9.5 × 10^−3^ Å^−1^ (**A**), q_z_ = 14.2 × 10^−3^ Å^−1^ (**B**), q_z_ = 18.7 × 10^−3^ Å^−1^ (**C**) and q_z_ = 21.0 × 10^−3^ Å^−1^ (**D**). The scanning step for the in-plane angle is Δφ = 10°, and therefore, the x-axis has 18 points. The XY reflectivity images were obtained using the image reconstruction of sinograms, based on a filtered-back projection algorithm. The images are displayed in the common logarithmic color scale. The contrast observed in the XY reflectivity images reveal the position and the shape of the buried metallic layers.

The experimentally collected data are 3 dimensional, and therefore, the data can be sorted as a series of sinograms, P_qz_(φ, u). Similar to computed tomography^22,23^, the reflectivity image R_qz_(x, y) is reconstructed from the sinogram P_qz_(φ, u). Figure 5 shows an example of sinograms (upper panel) and the XY reflectivity images (lower panel). The FBP algorithm^22–24^, which is one of the most widely used methods for image reconstruction, was employed in this investigation. At q_z_ = 9.5 × 10^−3^ Å^−1^ (Fig. 5A), the neutrons are totally reflected by the titanium/silicon (gold, nickel) interfaces, thus producing a homogeneous sinogram. Although the reflectivity ratio is high, the corresponding XY reflectivity image has a low quality because of the extremely low counting statistics in both Io and I_R_. However, the images for such lower q_z_ are usually not of main interest, because the inhomogeneity is evident at higher q_z_. In a sinogram, the feature from the sample forms a half period of a sine wave, and therefore, it is possible to determine the reflection contrast directly from sinograms. For the sinograms at q_z_ = 14.2 × 10^−3^ Å^−1^ (Fig. 5B), 18.7 × 10^−3^ Å^−1^ (Fig. 5C), and 21.0 × 10^−3^ Å^−1^ (Fig. 5D), it is possible to observe that the neutron reflectivity decreases rapidly as q_z_ increases. In the XY reflectivity images, almost all the nickel coated regions shown in Fig. 1A are visible at both 14.2 × 10^−3^ Å^−1^ (Fig. 5B) and 18.7 × 10^−3^ Å^−1^ (Fig. 5C). At 14.2 × 10^−3^ Å^−1^ (Fig. 5B), it is possible to observe some contrast at the position of the gold’s circle pattern. However, the triangle pattern is not very distinct. When comparing Figs. 5B and 5C, the rectangle nickel region and H-shape nickel regions resulted in different contrasts, indicating the difference in the thickness. At 18.7 × 10^−3^ Å^−1^ (Fig. 5D), the intensity is low, but it still clearly has information on the spatial distribution of the metallic coating in the sample.

## Discussions

### Line profiling in neutron reflectivity images

**Figure 6:**
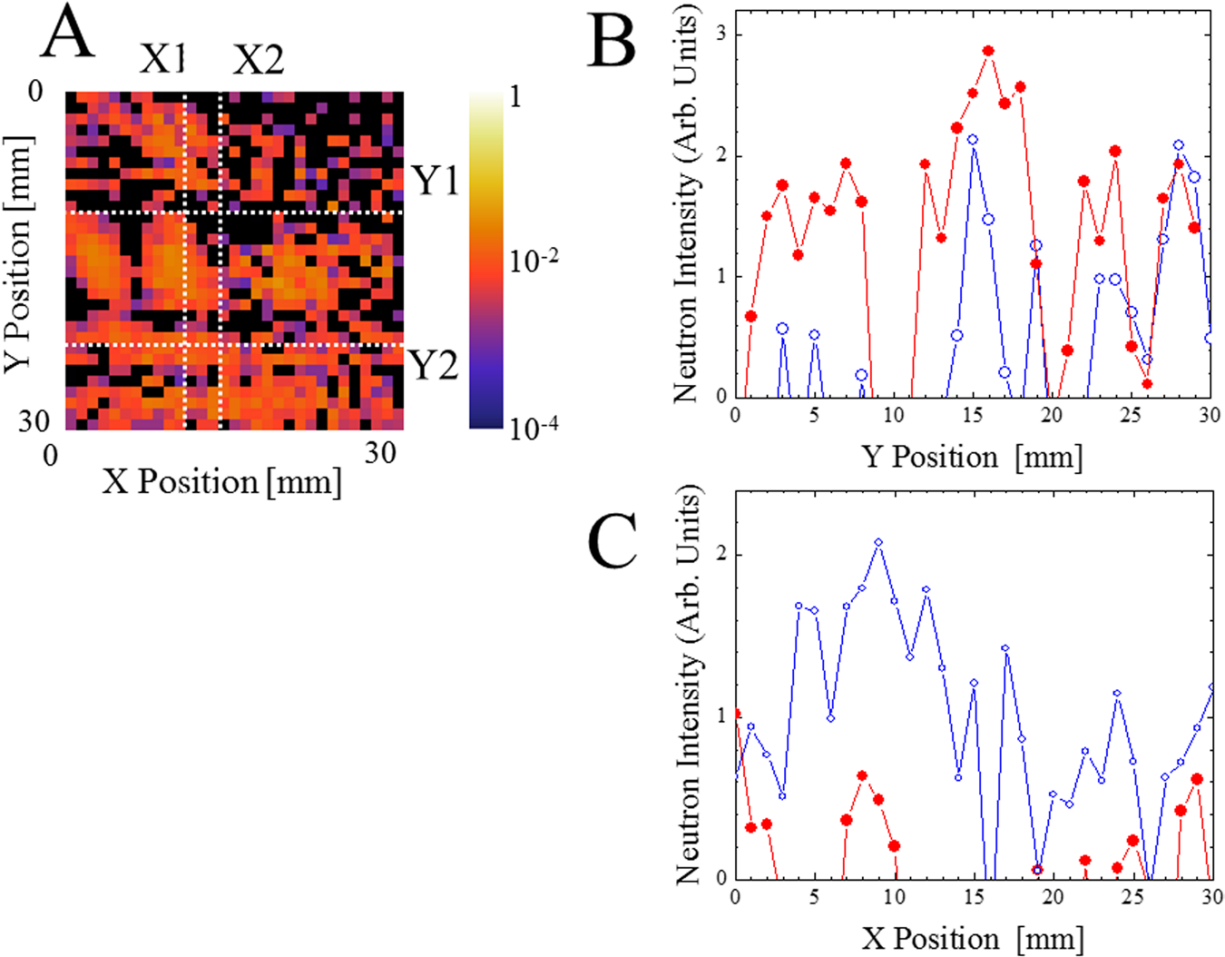
Example of line profile analysis (**B**,**C**) of the XY reflectivity image (**A**) for the patterned sample (schematically shown in Fig. 1A), at q_z_ = 13.5 × 10^−3^ Å^−1^. Line profile along the Y-axis (**B**): red closed circle  (X1, X = 10 and 11), blue open circle ○ (X2, X = 15 and 16), respectively. Line profile along X-axis (**C**): red closed circle ● (Y1, Y = 10 and 11), blue open circle  (Y2, Y = 22 and 23), respectively.

As the present method can facilitate the acquisition of XY reflectivity images, a line profile analysis would be informative when discussing the relationship between the layered structure and the physical properties of the thin film. Figure 6 shows some examples; B: line profiles along the Y direction, C: line profile along X direction for the image A (q_z_ = 13.5 × 10^−3^ Å^−1^). When comparing the Y-profiles (shown in Fig. 6B), X1 (X = 10 and 11) gives the profile corresponding to the gold circle, the nickel rectangle, two nickel lines, and the final nickel line, while X2 (X = 15 and 16) does not include the gold region. When comparing the X-profiles (shown in Fig. 6C), Y1 (Y = 10 and 11) identifies the no coating region, while Y2 (Y = 22 and 23) identifies the nickel line extending transversely. In the reconstructed data, one can obtain reflectivity for all points (in the present case, 31 × 31 points). The data are influenced by the statistical errors in counting the number of neutrons going through the Hadamard mask. In this profile analysis, the maximum reflectivity of around 10^−2^ is used to see the buried patterns. Therefore, the number of counts are limited, but one can still see clear distribution.

### Retrieval of local neutron reflectivity from video image sets

**Figure 7:**
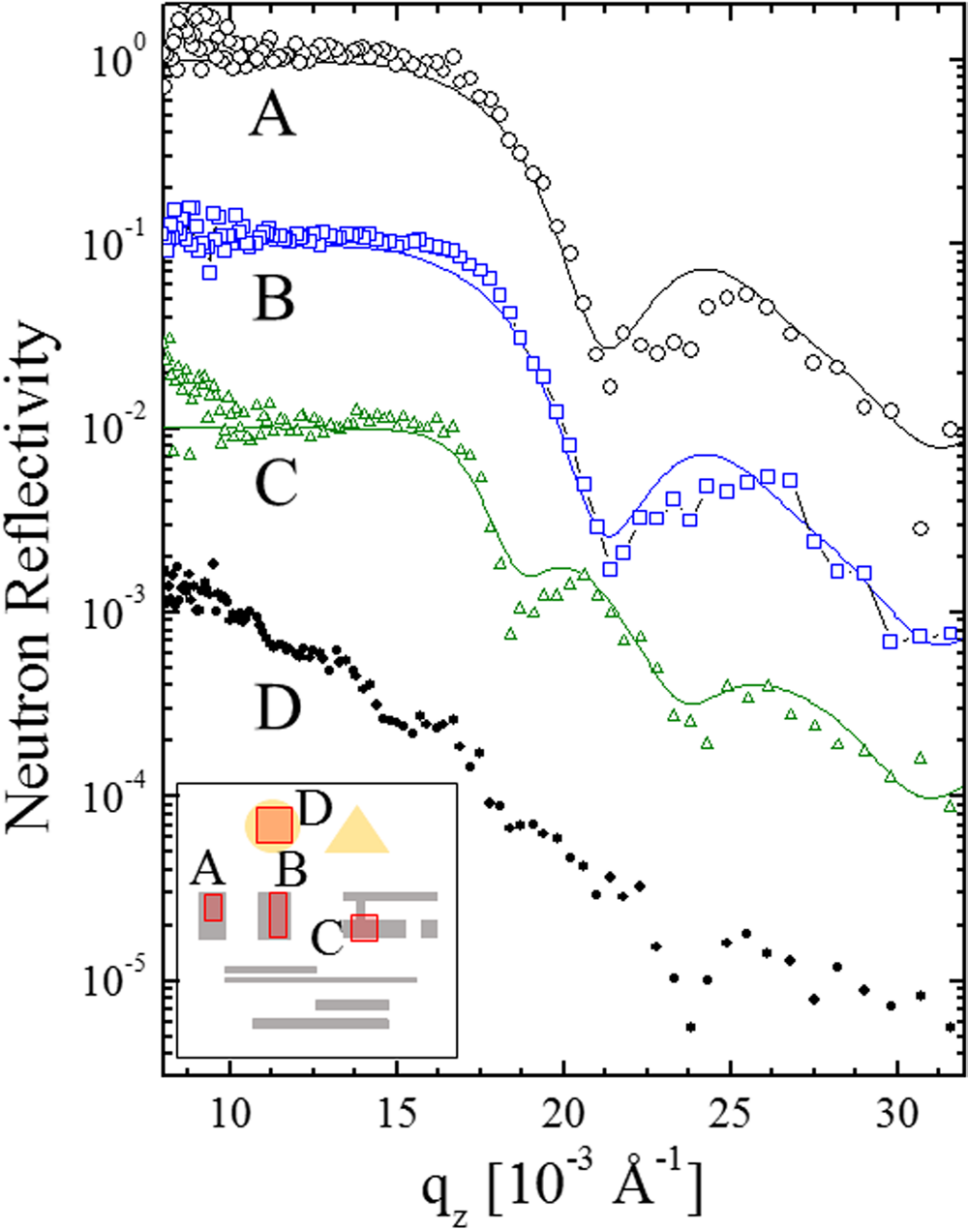
Selected neutron reflectivity profiles for specific regions of interests (ROIs) in the patterned sample (schematically shown in Fig. 1A). **A** (black open circle ○), **B** (blue open square □), **C** (green open triangle ) and **D** (black closed circle ●) correspond to the squares, of which left top and right bottom corners are the {(2, 14), (4, 17) nickel rectangle part close to the edge), {(10, 14), (12, 19) nickel rectangle part close to the center}, {(19, 17), (22, 20) nickel H-shape part}, and {(8, 3), (12, 7) gold circle part}, respectively. The positions for the above regions of interest (**A–D**) are shown schematically in the inset.

Because a series of neutron reflection intensity images sampled over a range of wavevector transfers are collected, a neutron reflectivity profile at the specific region of interest (ROI) can be extracted. Figure 7 shows some neutron reflectivity profiles extracted from reconstructed neutron reflection intensity images over the whole range of wavevector transfers q_z_ = 8~32 × 10^−3^ Å^−1^. While Fig. 7A (taken from the rectangle close to the edge) and 7B (taken from the rectangle close to the center) yielded similar reflectivity curves, the frequency of interference in Fig. 7C (taken from the H-shaped region) is apparently different. It was determined that the thickness of the two rectangle regions and the H-shaped regions are 490 Å and 745 Å, respectively, by fitting to the theoretical reflectivity. On the other hand, for Fig. 7D (taken from the gold circle region), it was not possible to get a reasonable fit, although a small dip is observed at approximately q_z_ = 14 × 10^−3^ Å^−1^, which is the critical q_z_ for gold. This could be due to some unexpected changes in the gold-coated regions after the deposition of the titanium protection layer. Though the gold/titanium interface is believed to be quite sharp and stable^29^, the present case might need to consider the influence from the side parts of the gold pattern, in addition to the top of the layer. The present imaging capability was able to give such information. The quality of the reflectivity data is influenced by the statistical errors in counting the number of neutrons going through the Hadamard mask. Although the area size for the regions of interest A, B, C and D are only 6~16 mm^2^, one could see clear features for each of them. Furthermore, it can be seen that the curves in Fig. 7 are not in agreement with the “averaged” neutron reflectivity shown in Fig. 1B, which is for the entire sample. This clearly indicates how the present imaging procedure helps the limitation of the conventional neutron reflectometry, which has a risk of wrong interpretation by “averaging” and neglecting inhomogeneity. One may consider that a conventional fitting scheme can be automatically applied for all (X,Y) points. As is often the case for the realistic thin films, data acquired by conventional reflectivity techniques might not be always easy to interpret. The data analysis based on fitting to a theoretically obtained reflectivity curve needs to be carefully performed when realizable models are not available. In general, a good fit does not automatically ensure the correct structure. In particular, the interpretation of the roughness of each interface is potentially problematic when the layer structure is not well-understood when the roughness is likely to be treated as mathematical parameters for obtaining better fits. In this case, examining the physical meaning of the obtained parameters is crucial, but inhomogeneity can result in some strange values. In this context, the imaging capability of the proposed method could be of great assistance. Therefore, the recommended scheme for the analysis is as follows: (1) analyze the inhomogeneity of the obtained video (the neutron reflection intensity and/or neutron reflectivity images as a function of q_z_), (2) identify groups of uniform area in the sample, (3) integrate the intensity in each area, (4) obtain neutron reflectivity curves for each group, and (5) perform fitting for each curve. The most important point is the ability of understanding inhomogeneity of layered structures in such a quantitative way. When several samples prepared under almost the same conditions show different properties (for example, optical or electronic), one would try to understand it by investigating the structures. In such cases, their structures are quite close, and the conventional “average” analysis just tells that the “average” structure is close each other. Visualizing inhomogeneity of the layered structures among such samples is already a great help for understanding the difference. For example, one could find some unexpected defects, pores and some discontinuity of layers etc. Then further analysis should be done for specific points, which are found through the above visualization. The present neutron reflectivity imaging technique can give such local information equivalent to the analysis by the use of small beam. Finally one would obtain much more reliable structure parameters than the conventional “average” analysis.

### Spatial resolution and measuring time in neutron reflectivity imaging

It is important to realize high-resolution imaging to evaluate inhomogeneity ranging from hundreds of microns to several nanometers. In the present research, a ~3 cm diameter area of the sample was analyzed as a 31 × 31 image with a spatial resolution of ~1 mm (as the opening slot of the Hadamard coded mask is 1 mm). The use of a multiwire proportional chamber with ^3^He gas (wire pitch, 1 mm) was tested in the earlier stage of the investigation. It was determined that the Hadamard coded mask resulted in superior reflection projection data for the same sample. In the future, parallel progress is expected, primarily dependent on the detector technology^30–32^; (i) the use of the a Hadamard coded mask with a greater number of smaller slots (such as 47 slots of 0.6 mm), (ii) the use of a high-resolution semiconductor pixel detector with a neutron sensitive layer coating (the spatial resolution can be better than 0.05 mm), and (iii) the combined use of the Hadamard coded mask and some position-sensitive neutron detector. For advanced imaging applications, improvement of the spatial resolution is crucial. This investigation is just a first step toward achieving this objective. In this case, the employed beam size and intensity are exactly the same as those in conventional neutron reflectivity measurements. The recorded neutron intensity at the detector is 50% on average because of the use of the Hadamard mask with a 50% opening. The use of a pixel detector could increase this value to 100%. It might also save the time necessary for the motion of the linear stage for the coded mask. When compared to other XY scanning methods that utilize a small beam, the advantages of the present technique become clear. Although the present method uses a primary neutron beam size of 0.2 mm (H) × 31 mm (V), it is possible to achieve a beam size of 0.2 mm (H) × 1 mm (V) by simply cutting and/or by focusing. However, truncating the beam leads to severe intensity loss (as ~30/31 will be lost), and focusing only partially helps. Furthermore, under a low angle incidence, the neutron beam illuminates ~30 mm (H) × 1 mm (V). To investigate a 1 mm (H) × 1 mm (V) area, it is necessary to shield the sample, which leads to a further intensity reduction at the detector. Moreover, XY scanning is necessary to acquire an image. If 31 × 31 points (as in the present work) are necessary, more than 900 reflectivity data points will be retrieved. Thus, it will be necessary to determine whether such a large number of measurements can be obtained for extremely low counts within a reasonable time. Therefore, in the case of neutrons, the data presented in this work is not easily obtained. In the proposed approach, the measuring time for one reflection projection was 272 s. The scanning points for the Hadamard coded mask and the in-plane angle is 31 × 18 = 558. The total acquisition time for one image was 42 h. Although a long time is required for the measurement, the observations of the samples that were made using this approach are otherwise very difficult or almost impossible to perform. Because the intensity of the neutron source in J-PARC will be increased by a factor of 5 in the future, it is expected that the measurement time will be substantially reduced.

### q_z_ range, q_z_ resolution, and sources of error in the reflectivity data

The q_z_ range was 8~38 × 10^−3^ Å^−1^, which is quite narrow and results in a limit of the reflectivity measurement to only 1–10^−4^ range. The main reason of this limitation is the restricted available spectral bandwidth of primary neutrons from the spallation source. The q_z_ range can be widened 2–3 times or even more by optimizing the time-interval (e.g., 60–80 ms instead of the current interval of 40 ms), the characteristics of the chopper (to minimize excessive exclusion of shorter wavelength neutrons), and the source-detector distance (e.g., 11–14 m instead of 16.3695 m) to capture reflections of slower neutrons. Another way to extend the q_z_ range is to acquire another image after changing the θ/2θ angles. In this case, the change of the footprint length should be taken into consideration. As in the case of conventional neutron reflectometry, the q_z_ resolution is limited by the angular divergence, i.e., combination of several narrow slits. It is not influenced by the Hadamard coded mask. It is also not influenced by the in-plane angle scanning, as long as careful adjustments are made so that the surface normal direction is parallel to the in-plane rotation axis. The Δq/q in the present work is approximately 5%. As the data are acquired by counting the number of neutrons of a specific wavelength corresponding to each q_z_, the main source of error is the statistical error in counting. It is known in computed tomography that the quality of the reconstructed image can be degraded by errors in the raw data. In the case of the present data, some images corresponding to lower the q_z_ part which yields a high reflectivity are distorted because of the errors associated with taking the ratio of small similar counts between the reflection and Io. In earlier experiments in X-ray reflectivity imaging, the data often included some ring artifacts caused by inhomogeneous responses of the 2D detectors^33,34^. Compared with this data, all reconstructed images shown in Fig. 5 are free from artifacts. One of the reasons is that the present system utilizes a point detector rather than a so-called pixel detector. The uniformity of the Hadamard coded mask is also perfect.

## Methods

### Sample description

The heterogeneous thin-film sample (schematically shown in Fig. 1A), is composed of shaped gold and patterned nickel layers under a homogeneous titanium layer. The sample was fabricated with an Eiko DID-5A magnetron sputtering system on a pre-cleaned silicon substrate (30 × 30 × 2 mm^3^). The neutron beam footprint fully covered the sample’s surface. The gold layers include the top-left circle (designed thickness ~400 Å) and top-right rectangle (designed thickness ~140 Å). The nickel layers consist of two rectangles (designed thickness ~500 Å), an H-shaped layer (designed thickness ~750 Å) and two bottom thin bars (designed thickness ~1000 Å).

### Neutron facility and the beamline instruments

The experiments were performed at the neutron reflectivity beamline BL17^35^, at the Materials and Life Science Experimental Facility (MLF) of J-PARC, Tokai, Ibaraki, Japan. The power of the proton accelerator was 200 kW during the present experiment. The neutron pulses through the coupled hydrogen moderator were triggered at a frequency of 25 Hz. The chopper system eliminates burst neutrons and avoids frame overlapping. The effective ToF range was determined to be 9.45~39.75 ms, which corresponds to the available neutron wavelength of 2.284~9.606 Å at the detector position at a distance of 16.3695 m from the source. This results in the corresponding q_z_ range of 7.991~33.6 × 10^−3^ Å^−1^ for the neutron reflectivity when 2θ is set at 0.7°. Eight independent XY slits (6 and 2 for the upstream and the downstream sides of the sample, respectively) are installed in the beamline, and the horizontal angular divergence is set at approximately 0.25 mrad, so that Δθ/θ is approximately 5% at 2θ = 0.7°. The beam size used was 0.2 mm (H) × 31 mm (V), and the footprint of the neutron beam on the sample was approximately 32.74 mm. Further details of the slit system at BL17 are provided elsewhere^35^.

### Neutron reflectivity imaging setup

The sample stages were prepared at the National Institute for Materials Science, Tsukuba Japan and were transferred to the BL17, J-PARC MLF. The setup is equipped with two translational stages, one in-plane rotation stage with tilt-adjustments stages in both the X and Y directions, a single-axis high-resolution goniometer, and the sample support with a vacuum chuck. One translational stage adjusts the surface of the sample to the center of the neutron beam, while another translational stage is employed to bring the goniometer’s rotation center to the center of the neutron beam. The goniometer is a compact Kohzu Tangent-bar Rotary Stage dedicated to grazing incidence experiments, with a stroke of ±2.5° and a resolution better than 1/90,000°. The in-plane rotational motor scans the in-plane angle from 0° to 180° with a resolution of 0.1°. Because it is important to set the surface normal direction exactly on the rotational axis, two tilt adjusters are used. The source to the sample distance is 15.5 m, which is the standard condition at the beamline. The sample to the ^3^He detector distance was 0.8695 m. A Hadamard coded mask is a new addition to the beamline instrument. The mask is positioned just in front of the ^3^He detector, which has a window of 150 × 25.4 mm^2^.

### Data handling

All neutron detection events in the ^3^He detector are stored with timestamps relative to the trigger of their associated neutron pulses. The time information is recorded as an integer between 0 and 2^24^ (16777216), and the ToF is obtained by multiplying by 25 ns. The events were sorted out as a histogram according to the ToF and arranged by integrating 12,000 data points so that the width is 0.3 ms, considering the counting statistics of the experiment. The number of ToF histogram data converted from such event records for one sample is 558 (=31 × 18). Initially, the data are grouped by 18 (φ angle). Then one group includes 31 raw files that are Hadamard encoded. After decoding and normalization by the Io profile, each group yields one reflectogram. By reorganizing the data in the reflectograms, many sinograms (φ angle vs. position, for each q_z_) are obtained. The next step is image reconstruction from the sinograms. In the present work, the FBP method^22–24^ was employed. Then a real-space XY reflectivity map is obtained for each q_z_. Finally, the line profile analysis and neutron reflectivity analysis for each selected ROI is performed. To facilitate the processing of all datasets based on the described procedure, proprietary software was prepared at the National Institute for Materials Science, Tsukuba, Japan.

## Supplementary information

Video of XY neutron reflectivity images as a function of qz

Video of XY neutron reflectivity images as a function of qz

## Data Availability

All neutron data collected in the present research are stored as binary event data in the public storage system in the BL17, J-PARC MLF, Ibaraki, Japan. In principle, all data can be disclosed to any third parties if the facility approves.
