# Supplementary material for: Neutron visualization of inhomogeneous buried interfaces in thin films
Source: Sci Rep. 2019 Jan 24;9:571. doi: 10.1038/s41598-018-37094-5 (PMC6345982; doi:10.1038/s41598-018-37094-5)
Supplement: Supplementary file 2 — Video of XY neutron reflectivity images as a function of qz [file 41598_2018_37094_MOESM2_ESM.pdf]

Supplementary Information

Neutron visualization of inhomogeneous buried interfaces in thin films

Kenji Sakurai[1,2,a)], Jinxing Jiang[2,1)], Mari Mizusawa[3,1)], Takayoshi Ito[3)], Kazuhiro Akutsu[3)], Noboru Miyata[3)]

[1]*National Institute for Material Science, 1-2-1, Sengen, Tsukuba, Ibaraki, 305-0047, Japan*
[2]*University of Tsukuba, 1-1-1, Tennodai, Tsukuba, Ibaraki, 305-0006, Japan*
[3]*Comprehensive Research Organization for Science and Society (CROSS), Tokai, Ibaraki 319-1106, Japan*

a) Corresponding Author, sakurai@yuhgiri.nims.go.jp

S1. Video of XY neutron reflectivity images as a function of q_z

Similar to computed tomography, which gives cross-sectional image by handling a set of transmission images, the present method gives neutron reflectivity image from the sinogram, i.e., the reflection projection taken as a function of in-plane rotation angles. In the present work, well-known filtered back projection (FBP) has been employed for the image reconstruction. The obtained image has 31 × 31 pixels, which concerns the number of slots in the employed Hadamard mask (31 slots). The number of projections used for the image-reconstruction is 18. The number of images in the movie corresponds to the band of q_z range. As seen in the video, one can see the buried patterns become visible by scanning q_z, and the variety of q_z dependence corresponds to inhomogeneity of the film structures such as thickness, density (different materials) and roughnesses.
